# Correction: Theoretical investigations of the electronic and optical properties of a GaGeTe monolayer

**DOI:** 10.1039/d3ra90064a

**Published:** 2023-07-14

**Authors:** Nguyen Thi Han, Vo Khuong Dien, Tay-Rong Chang, Ming-Fa Lin

**Affiliations:** a Department of Physics, National Cheng Kung University 1 University Road Tainan 70101 Taiwan han.nguyen.dhsptn@gmail.com u32trc00@phys.ncku.edu.tw; b Center for Quantum Frontiers of Research and Technology (QFort) Tainan 70101 Taiwan; c Physics Division, National Center for Theoretical Sciences Taipei 10617 Taiwan; d Hierarchical Green-Energy Materials (Hi-GEM) Research Center, National Cheng Kung University Taiwan

## Abstract

Correction for ‘Theoretical investigations of the electronic and optical properties of a GaGeTe monolayer’ by Nguyen Thi Han *et al.*, *RSC Adv.*, 2023, **13**, 19464–19476, https://doi.org/10.1039/D3RA03160H.

The authors regret that the Acknowledgments section was omitted from the original article. Acknowledgments are as shown below.

## Acknowledgments

M.-F. L. was supported by the Hierarchical Green-Energy Materials (Hi-GEM) Research Center, from the Featured Areas Research Center Program within the framework of the Higher Education Sprout Project by the Ministry of Education (MOE) and the Ministry of Science and Technology (MOST 111-2112-M-006-020) in Taiwan.

T.-R. C. was supported by the 2030 Cross-Generation Young Scholars Program from the National Science and Technology Council (NSTC) in Taiwan (Program No. MOST111-2628-M-006-003-MY3), National Cheng Kung University (NCKU), Taiwan, and National Center for Theoretical Sciences, Taiwan. This research was supported, in part, by the Higher Education Sprout Project, Ministry of Education to the Headquarters of University Advancement at NCKU.

The Royal Society of Chemistry apologises for these errors and any consequent inconvenience to authors and readers.

## Supplementary Material

